# Acute Vasitis clinical picture mimicking inguinal hernia: Case report and review

**DOI:** 10.1016/j.eucr.2021.101847

**Published:** 2021-09-10

**Authors:** Mohammed Almutairi, Hatem Althubiany, Ahad Alhabsi, Kamel fadaak, Mona Almuhaish, Faisal Albalawi

**Affiliations:** a Department of Urology, Imam Abdulrahman Bin faisal University, Saudi Arabia; b Department of Radiology, Imam Abdulrahman Bin faisal University, Saudi Arabia

**Keywords:** Acute vasitis, Inguinal hernia, Acute scrotal pain

## Abstract

Acute Vasitis a rare condition, but one that can present with a diagnostic dilemma, if not recognized and managed appropriately, may lead to unnecessary surgical interventions with consequent morbidity for the patient. A 27 year old healthy male, presented with left scrotal pain associated with inguinoscrotal swelling.

Physical examination revealed left scrotum tenderness and swelling extended along left inguinal area. CT showed multi loculated cystic fluid collection within left seminal vesicle. Acute Vasitis is a rare differential diagnosis for acute scrotum and This case report summarizes the importance of identifying it and how imaging can prevent unnecessary surgical intervention.

## 1 Introduction

Vasitis is a rare entity characterized into acute painful infectious Vasitis and a symptomatic Vasitis nodosa by Chen and Schlegel.^1^

Acute vasitis is a rare condition, but one that can be a diagnostic dilemma, and if not recognized and managed appropriately, may lead to unnecessary surgical interventions with consequent morbidity for the patient.^2^ We reviewed the cases in Literature and we identified 91 well-documented cases before our case have been reported so far since the first case in 1943^1^

## 2 Case report

This is a 27-year-old male not known to have any medical illnesses presented to the emergency department complaining of severe left scrotal pain and swelling that started 4 days prior to his presentation. The pain developed after the patient fell down, then progressively increased in intensity, radiating to the left inguinal area, and associated with inguinoscrotal swelling, nausea and one episode of gross hematuria, patient denied having fever or lower urinary tract symptoms, there was no history of sexual intercourse, previous similar complain and no past surgical history. upon examination the patient was vitally stable, the left scrotum was severely tender and swollen, along with tender mass over the left inguinal area. WBC was 18,000 and other labs were within normal.

## 3 Scrotal ultrasound

**Fig. 1:**
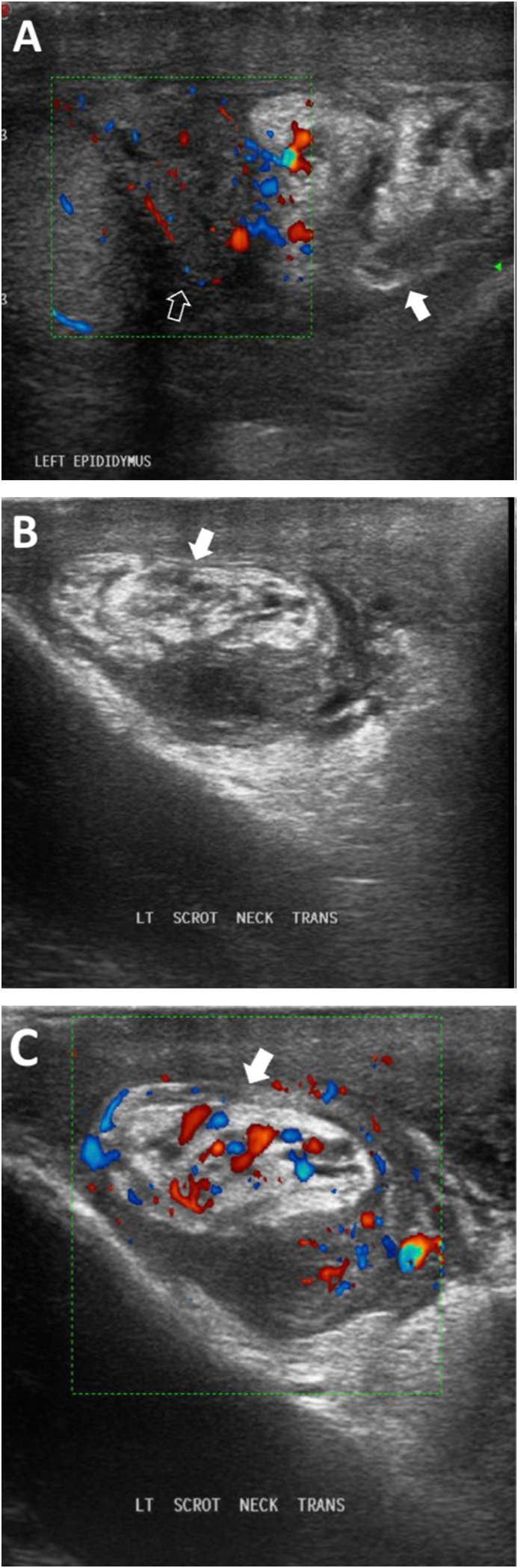
(A,B , C) Gray scale and color Doppler US images showing heterogenous echogenicity lesion (solid arrow) adjacent to the head of left epididymis (empty arrow) with increased vascularity of both. (For interpretation of the references to color in this figure legend, the reader is referred to the Web version of this article.)

Testicular ultrasound showed slightly increased vascularity on the left testis and epididymis with picture of query incarcerated inguinal hernia. therefore, the general surgery tram was consulted. CT abdomen was ordered by general surgery as shown in (Fig .1).

## 4 Contrast-enhanced CT scan of the abdomen and pelvis

**Fig. 2:**
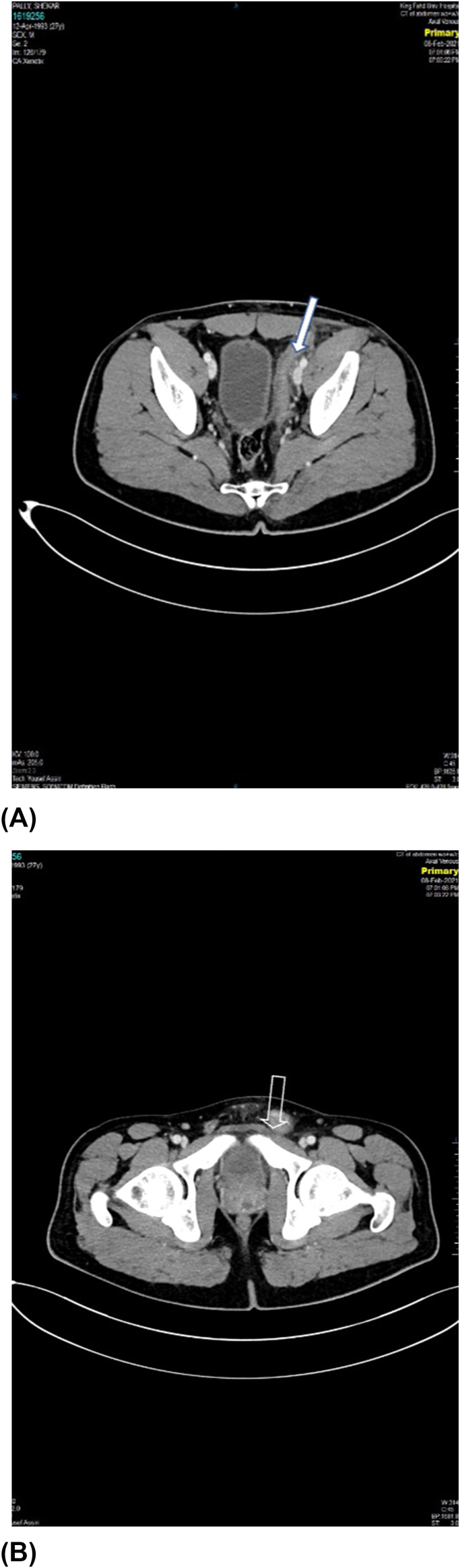
(A , B) Contrast enhanced CT scan axial images showing significantly thickened and swollen left spermatic cord (empty arrow)and vas deference (solid arrow) with surrounding inflammatory fat strandings. A small rim enhancing collection (arrow head) is seen in the most inferior aspect of the left seminal vesicle/left ejaculatory duct measuring 1.5*2.8 mm in maximum dimension.

**Fig. 3:**
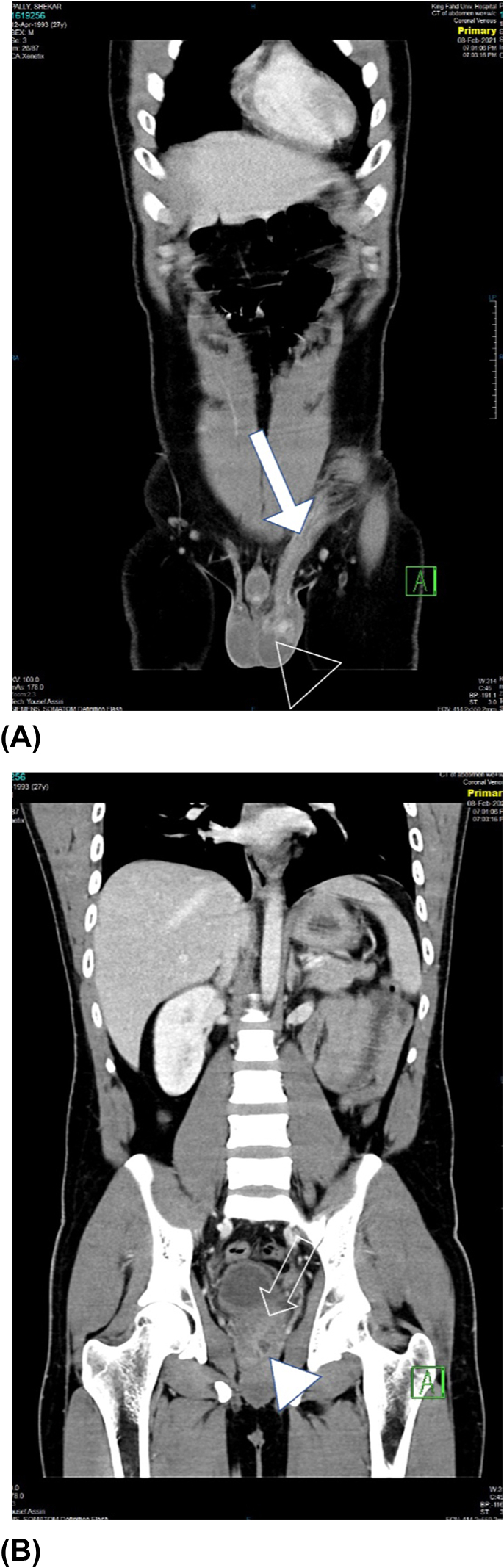
(A, B) Contrast enhanced CT scan coronal images showing hyperenhancement of the left epididymal head (empty arrow head) with vascular engorgement, swelling and significant thickening of the left spermatic cord (solid arrow). This inflammation is seen extending more proximally along the left inguinal canal. The left seminal vesicle (empty arrow) showing increase enhancement with a small rim enhancing collection (arrow head) in its most inferior aspect measuring 1.5*2.8 mm in maximum dimension.

Revealed hyperenhancement of the left epididymal head with vascular engorgement, significant thickening, swelling, and fat strandings of the left spermatic cord. This inflammation is seen extending more proximally along the left inguinal canal to involve the vas deference as well as left seminal vesicle which showed increase enhancement compared to the contralateral side. In addition, a small collection was identified in the most inferior aspect of the left seminal vesicle/left ejaculatory duct showed peripheral rim enhancement measuring 1.5*2.8 mm in maximum dimension. Epididymitis, funiculitis and vasitis with small seminal vesical abscess as shown in (Fig. 2, Fig. 3) patient was treated with oral antibiotics ceftriaxone IM one dose, levofloxacin 14 days. patient improved significantly after 2 weeks and symptoms disappeared.

## 5 Discussion

Vasitis nodsa was first described in 1943 by Benjamin as asymptomatic, chronic inflammation associated with obstruction of vas deference which leads to increase intra-luminal pressure with spermatozoa leakage and inflammatory process.^3^

The ascending spread of urinary pathogens such as H. influenza and *E. coli* is thought to be the case of Acute infective Vasitis with negative culture in most of the cases.^3^

Clinical symptoms include asymptomatic nodular lesion in vas deference and are most common in patients following vasectomy.^3^

Patients with acute Vasitis may present with localized pain or palpable mass and \ or swelling in the scrotal or inguinal area some of them associated with fever and leukocytosis.^3^

It can be easily confused with other deferential diagnosis such as strangulated inguinal hernia, epididymo-orchitis and or even testicular torsion.^3^

Usually the diagnosis is unclear, and CT is frequently needed. CT findings include vas deferens thickening, spermatic cord edema and peripheral fat stranding. Literature support the use of CT or MRI to clarify the anatomy and to role out incarcerated inguinal hernia.^4^

Acute Vasitis is often diagnosed intra operatively. In our case to the radiologist had a high index of suspicion and requested for a complementary computerized tomography scan to clarify any doubts^5^

## 6 Conclusion

Clinical and ultrasound findings of Vasitis, epididymitis, orchitis and inguinal hernia could share some similarities. Further radiologic evaluation by CT Scan is recommended to distinguish between them and avoid unnecessary surgical exploration.

## Financial support and sponsorship

None.

## Declaration of competing interest

No conflicts of interest.

## References

[1] Sultan A., Hassan M., Choudhry M. (2021). Vasitis nodosa: a rare diagnosis for inguinal swelling. Cureus.

[2] Patel K., Lamb B., Pathak S., Peters J. (2014). Vasitis: the need for imaging and clinical acumen. BMJ Case Rep.

[3] Chen C.W., Lee C.H., Huang T.Y., Wang Y.M. (2019 Apr 29). Vasitis: a rare diagnosis mimicking inguinal hernia: a case report. BMC Urol.

[4] Bomar A.J., Epelman M.S., Ellsworth P.I. (2020 Jul 29). Acute vasitis presenting as a concerning paratesticular mass in an adolescent, a case report. Urol Case Rep.

[5] Akinola Rachael A. (2017). Acute vasitis presenting as an inguinoscrotal swelling: a diagnostic dilemma. Int J Clin Case.

